# Association of High Blood Pressure With Physical Activity, Screen-Based Sedentary Time, and Sedentary Breaks in a 2-Year Cohort of Community Dwelling Adults

**DOI:** 10.3389/ijph.2022.1605139

**Published:** 2022-09-20

**Authors:** William R. Tebar, Gregore Iven Mielke, Raphael M. Ritti-Dias, Kelly Samara Silva, Daniel S. Canhin, Catarina C. Scarabottolo, Jorge Mota, Diego G. D. Christofaro

**Affiliations:** ^1^ Movement Science Post-graduation Program, Universidade Estadual Paulista–Unesp, Presidente Prudente, Brazil; ^2^ School of Public Health, The University of Queensland, Brisbane, QLD, Australia; ^3^ Post-graduation Program in Rehabilitation Science, Universidade Nove de Julho–UNINOVE, Sao Paulo, Brazil; ^4^ Post-Graduation Program in Physical Education, Universidade Federal de Santa Catarina–UFSC, Florianopolis, Brazil; ^5^ Faculdade de Desporto–FADEUP, Centro de Investigação em Atividade Física, Saúde e Lazer (CIAFEL), Universidade do Porto, Porto, Portugal

**Keywords:** sedentary behavior, epidemiology, hypertension, lifestyle habits, cardiovascular risk factor

## Abstract

**Objective:** This study analyzed the association of high blood pressure (HBP) with physical activity, sedentary behavior, and sedentary breaks in 2-year follow-up.

**Methods:** A sample of 331 middle-aged and older adults (mean age of 59.6 ± 17.3 years) was randomly selected and assessed at baseline and after 2 years of follow-up. HBP was considered as ≥140/90 mmHg values of systolic and diastolic blood pressure. Physical activity, sedentary behavior, and sedentary breaks were assessed by questionnaire. Age, sex, socioeconomic status, and body mass index were covariates.

**Results:** Continuous HBP was observed in 26.3% of sample between baseline and follow-up. Adults who reported continuously high sedentary breaks at leisure activities were less likely to have HBP (OR = 0.34, *p* = 0.011), as well as those who remained high physically active (OR = 0.41, *p* = 0.016), even after mutual adjustment. No association was observed between high sedentary behavior and HBP at follow-up.

**Conclusion:** Community dwelling adults who were high active and performed frequent sedentary breaks were less likely to have HBP in 2-year follow-up. Strategies for HBP control should considered both physical activities and leisure sedentary breaks in adult population.

## Introduction

High blood pressure (HBP) is defined as above-normal pressure in blood vessels when heart muscles contract (systolic blood pressure) and/or when heart muscles relax (diastolic blood pressure), being these above-normal cutoff points defined as 140 and 90 mmHg (millimeters of mercury) values, respectively [1]. HBP is considered as the leading global burden for cardiovascular disease [2], being responsible for about half of strokes and ischemic heart diseases, as well for 7.6 million of premature deaths worldwide [3].

The risk of having HBP increase in the aging process, which has been associated to conditions of inflammation, oxidative stress and endothelial dysfunction [4]. In regard sex, higher HBP risk was observed in men than women [5], which is affected by the different hormonal, physiological, and behavioral profiles [6, 7]. It has been also reported that low socioeconomic status was associated to higher blood pressure [8].

Besides these sociodemographic factors, the lifestyle habits have been widely associated to HBP in adult population. The physical activity practice provides positive adaptations on cardiovascular health which may contributes to reduce HBP risk, as improvement on cardiovascular function [9] and increase in energy expenditure to weight control [10]. Otherwise, sedentary behavior was associated to risk of hypertension incidence [11] and has been associated to higher adiposity levels [12].

The lifestyle habits are considered as an important modifiable risk factor for blood pressure control in epidemiological scope [13], and healthy habits as physical activity engagement and lower sedentary behavior across the time may be an important contributor for blood pressure control in adult population. In this sense, the objective of this study was to analyze the association of physical activity, screen-based sedentary time and sedentary breaks with HBP in middle-aged and older adults after 2 years of follow-up.

## Methods

### Study Design

This is a 2-year observational longitudinal study involving a randomly selected sample of adults with 18 and more years of age. This research was previously approved by the Sao Paulo State University Research Ethics Committee, at protocol CAAE 45486415.4.0000.5402. An Informed Consent Term was signed by all participants, with explanation about study procedures, guarding personal information and the possibility to desert at any time.

### Sampling Process

The study was conducted in the city of Presidente Prudente, which has a population of 176,124 adults and is located in in the southeastern region of Brazil. The baseline sample was selected by dividing the city into five geographical regions (central, north, south, east, and west). The streets of each region were listed and randomly selected and all the households of each selected street were visited. At door to door, the researchers asked for adults with 18 years of age and more, which agreed to take part in the study and performed the research procedures at the household of participant, in a single day. The baseline sampling process was reported in a previous study [14].

For the present study, the households were visited after 2 years of the first assessment, where was performed the same evaluations. A sample of 449 participants were contacted and enrolled in this longitudinal wave, where 105 participants gave up during the assessment period (23.4%), 7 participants were unable to participate (1.5%), and 6 participants died in the period between cross-sectional and longitudinal stages (1.3%). At the end, a total of 331 participants were assessed (73.7%), as reported in previous study [15]. The data collection occurred between April 2016 and October 2019.

### Blood Pressure Measurement

A digital oscillometric device (OMRON^®^ brand, model HEM-4200) was used to the assessment of blood pressure values, previously validated to adult population [16]. The measurement was performed with the participant in seated position, resting for 10 min for the first collect and with a second measure collected 5 min later. The mean value of the two measures for systolic and diastolic values was calculated. The cutoff point of ≥140/90 mmHg was used to classify the sample with high blood pressure [17].

### Physical Activity Engagement

The questionnaire of Baecke et al. [18] was used to assess the habitual practice of physical activity of the sample. This instrument was previously validated to Brazilian adult population [19] and against gold-standard methods, such as doubly-labelled water [20]. This instrument assesses habitual practice of physical activity in the domains of work/occupation, sport, and leisure time/commuting, through 16 questions in a Likert scale, providing a dimensionless score from 1 to 5 for each assessed domain. At the end, the three scores were summed in order to obtain the total physical activity score. Due to the lack of cutoff points from Baecke questionnaire to determine physically actives, those participants who were located at the 4th quartile of total physical activity score were classified as high actives, while those who were in the lower quartiles (1st, 2nd, and 3rd) were classified as less actives.

### Screen-Based Sedentary Time

Information about self-reported daily hours spent in screen devices was considered to assess screen-based sedentary time of the sample. This instrument was used in previous studies among adult population [21, 22]. In the present study, it was considered the sum of hours spent in TV viewing, computer and cell phone use for a typical weekday and for a typical weekend day. The total screen-based sedentary time was obtained by the calculation of the mean hours between the weekday and weekend day. The cutoff point of with 8 and more hours proposed by KU et al. [23] was used to classify the sample as high or low screen-based sedentary time.

### Breaks in Sedentary Time

The sedentary breaks were assessed by the frequency of interruptions in sedentary behavior at leisure time, as previously used [24–26]. This study used a five-point Likert scale ranged as “never”, “rarely”, “sometimes”, “often”, and “always” for the interruptions in sitting/lying position through the question: “At your leisure-time in a typical day, how frequently do you interrupt your sedentary time by standing or walking for at least 1 min, whether to drink water, go to the bathroom, or doing other activities without sitting or lying?” Participants were classified as low sedentary breaks (never, rarely, sometimes) and as high sedentary breaks (often, always) [27].

### Body Mass Index

Objective measurements of body mass (in kilograms) and height (in meters) were used to calculate body mass index (BMI = kg/m^2^). Body mass was assessed by digital scale with capacity of 180 kg and precision in 0.1 kg (Omron HBF-514, Omron Healthcare Co. Ltd., Kyoto, Japan), while height was collected through a portable stadiometer (Seca 213, Seca GmBH and Co. Kg, Hamburg, Germany), with maximum capacity of 2.2 m and precision in 0.1 cm. The measurements were collected with the subjects barefoot and with light clothing. The body mass index was classified according to global recommendations: normal weight between 18.5 and 24.9 kg/m^2^, overweight between 25.0 and 29.9 kg/m^2^, and obesity as 30.0 kg/m^2^ or more [28].

### Socioeconomic Status

The Brazilian Criteria for Economic Classification [29] was used to assess the socioeconomic status of the sample. This instrument considers the educational level and specific rooms and consumer goods in the household, classifying the sample into the socioeconomic classes A, B1, B2, C1, C2, D-E, from the highest to the lowest, which were further categorized into high (A), medium (B1, B2, C1), and low (C2, D-E) socioeconomic class.

### Statistical Analysis

Sample characterization is presented in frequency and 95% confidence interval. Comparison between proportions at baseline and follow-up was performed by chi-square test. Means comparison was performed by independent sample t-test when grouping variables had two categories and by analysis of variance with post hoc of Bonferroni when grouping variables had three or more categories. Cross-sectional association between HBP and independent variables was analyzed by multiple models of binary logistic regression adjusted by sex, age, socioeconomic status, and body mass index. The association of HBP with the cluster of behaviors from baseline to follow up was analyzed by multiple models of binary logistic regression simultaneously adjusted by HBP at baseline, and total sedentary behavior, physical activity score, and frequency of sedentary breaks at follow-up. For this model, those categories of behaviors which remained the same from baseline to follow up were named as “continuously”. Statistical significance was considered at *p* < 0.05 level and confidence interval of 95%, with analysis performed through SPSS^®^ Statistical Package version 24.0.

## Results

This study assessed a total of 331 participants. Persistent HBP was observed in 26.3% of the sample after 2 years of follow-up. No difference in sex proportions was observed according to categories of independent variables. Regarding the age, participants with high sedentary breaks and with high level of screen-based sedentary time were significantly younger than their counterparts at both baseline and follow-up (*p* < 0.001). The characterization of sample is presented in Table 1.

**TABLE 1:** Characteristics of sample (Presidente Prudente, Brazil. 2022).

	Baseline				Follow-up			
	n	%	Age	% Sex	n	%	Age	% Sex
			Mean (SD)	Males/Females			Mean (SD)	Males/Females
Overall sample	331	100.0	59.6 (17.3)	31.7/68.3	331	100.0	61.6 (17.2)	31.7/68.3
Males	105	31.7	59.3 (19.2)	—	105	31.7	61.1 (19.0)	—
Females	226	68.3	59.7 (16.4)	—	226	68.3	61.8 (16.3)	—
Body mass index^a^								
Normal weight (18.5–24.9 kg/m^2^)	107	32.3	58.3 (19.8)	29.0/71.0	95	28.7	60.4 (20.9)	28.4/71.6
Overweight (25.0–29.9 kg/m^2^)	120	36.3	59.7 (16.4)	35.0/65.0	128	38.7	61.7 (15.9)	34.4/65.6
Obesity (≥30.0 kg/m^2^)	104	31.4	60.7 (15.6)	30.8/69.2	108	32.6	62.4 (14.9)	31.5/68.5
Socioeconomic level								
High	68	20.5	55.6 (17.9)	33.8/66.2	68	20.5	57.7 (17.9)	33.8/66.2
Medium	247	74.6	60.4 (16.9)	31.6/68.4	247	74.6	62.4 (16.7)	31.6/68.4
Low	16	4.8	63.8 (19.8)	25.0/75.0	16	4.8	65.5 (19.9)	25.0/75.0
Sedentary breaks								
Low breaks	96	29.0	67.6 (15.2)	35.4/64.6	96	29.0	69.4 (15.2)	35.4/64.6
High breaks	235	71.0	56.3 (17.0) ^b^	30.2/69.8	235	71.0	58.4 (16.9) ^b^	30.2/69.8
Screen-based sedentary time^a^								
Low level	274	82.8	63.2 (15.4)	31.0/69.0	226	68.3	67.1 (14.4)	33.2/66.8
High level	57	17.2	42.1 (15.4) ^b^	34.6/65.4	105	31.7	48.1 (16.3) ^b^	29.5/70.5
Physical activity								
Less active	238	71.9	60.8 (17.8)	31.5/68.5	247	74.6	62.8 (17.5)	32.8/67.2
High active	93	28.1	55.9 (15.6)	33.3/66.7	84	25.4	57.4 (15.7)	29.6/70.4

a Statistical significance by chi-square test for comparison of proportions at *p* < 0.05 level between baseline and follow-up.

b Statistical significance between categories at the same moment (baseline or follow-up). CI, confidence interval; Low breaks = never, rarely, sometimes; High breaks = frequently and always; Low level = Less than 8 h/day; High level = 8 and more hours/day; Less active = 1st, 2nd, and 3rd quartiles of Baecke score; High active = 4th quartile of Baecke score.

The 105 participants who gave up during the follow-up were 58.1% of females (sex proportions were marginally different when compared to those who were included, chi-square test *p* = 0.055) and presented an average age of 56.5 ± 22.1 years, being slightly younger than those who were included in this study (independent samples t test *p* < 0.001).

Table 2 contains the cross-sectional association between HBP and independent variables. Adults with high sedentary breaks were 44% and 46% less likely to have HBP at baseline and follow-up respectively, when compared to those with low sedentary breaks (Baseline OR = 0.56, *p*-value = 0.035; Follow-up OR = 0.54, *p*-value = 0.027), regardless of sex, age, socioeconomic status, and body mass index. No other cross-sectional association was observed.

**TABLE 2:** Association of high blood pressure at baseline and follow-up with sedentary behavior, sedentary breaks, and physical activity in middle-aged and older adults (Presidente Prudente, Brazil. 2022).

	High blood pressure							
	Baseline				Follow-up			
	OR	95% CI	*p*-value	H&L	OR	95% CI	*p*-value	H&L
Sedentary breaks								
Low breaks	1.00	Reference	—		1.00	Reference	—	
High breaks	0.56	0.32; 0.96	0.035	0.576	0.54	0.32; 0.93	0.027	0.992
Screen-based sedentary time								
Low level	1.00	Reference	—		1.00	Reference	—	
High level	0.69	0.29; 1.65	0.406	0.160	0.92	0.48; 1.76	0.804	0.812
Physical activity								
Less active	1.00	Reference	—		1.00	Reference	—	
High active	0.83	0.47; 1.46	0.528	0.169	0.78	0.43; 1.39	0.402	0.772

Analysis adjusted by age, sex, socioeconomic status, and body mass index at baseline and follow-up, respectively. OR, odds ratio; CI, confidence interval; Low breaks = never, rarely, sometimes; High breaks = frequently and always; Low level = Less than 8 h/day; High level = 8 and more hours/day; Less active = 1st, 2nd, and 3rd quartiles of Baecke score; High active = 4th quartile of Baecke score; H&L = *p*-value of Hosmer & Lemeshow test for model fit (values higher than 0.05 have a good fit).

Table 3 shows the association of HBP with screen-based sedentary time, sedentary breaks, and physical activity at follow-up. Those adults who reported high sedentary breaks at baseline and follow-up were 66% less likely to have HBP than those with low sedentary breaks (Odds ratio = 0.34, *p*-value = 0.011). Adults who remained high physically actives from baseline to follow-up presented 59% less chance of having HBP when compared to those adults who were continuously less active from baseline to follow-up (Odds ratio = 0.41, *p*-value = 0.016). The association between screen-based sedentary time and HBP lost its significance in adjusted analysis.

**TABLE 3:** Association of high blood pressure with screen-based sedentary time, sedentary breaks, and physical activity from baseline to follow-up in middle-aged and older adults (Presidente Prudente, Brazil. 2022).

	High blood pressure at follow-up						
	Adjusted by high blood pressure at baseline			Adjusted for mutual behaviors^a^			
	OR	95% CI	*p*-value	OR	95% CI	*p*-value	H&L
Sedentary breaks							
Continuously low (*n* = 96)	1.00	Reference	—	1.00	Reference	—	0.999
Continuously high (*n* = 235)	0.36	0.20; 0.64	0.001	0.34	0.15; 0.78	0.011	
Screen-based sedentary time							
Continuously high (*n* = 105)	1.00	Reference	—	1.00	Reference	—	0.807
High to low (*n* = 21)	0.29	0.09; 0.93	0.038	0.87	0.39; 1.91	0.727	
Low to high (*n* = 77)	0.42	0.21; 0.87	0.019	0.67	0.12; 3.63	0.644	
Continuously low (*n* = 128)	0.43	0.23; 0.81	0.009	0.41	0.16; 1.07	0.068	
Physical activity							
Continuously less active (*n* = 238)	1.00	Reference	—	1.00	Reference	—	0.283
Less to high active (*n* = 0)	—	—	—	—	—	—	
High to less active (*n* = 9)	0.73	0.15; 3.53	0.698	0.72	0.14; 3.68	0.696	
Continuously high active (*n* = 84)	0.45	0.24; 0.86	0.016	0.41	0.21; 0.83	0.016	

a Analysis adjusted by high blood pressure at baseline, sedentary breaks, total screen-based sedentary time, and physical activity score at follow-up. OR, odds ratio; CI, confidence interval; High sedentary behavior = 8 and more hours/day; Low sedentary behavior = Less than 8 h/day; High active = 4th quartile of Baecke score; Less active = 1st, 2nd, and 3rd quartiles of Baecke score; Continuously = remained from baseline to follow-up; H&L = *p*-value of Hosmer and Lemeshow test for model fit (values higher than 0.05 have a good fit).

## Discussion

The main findings of this study showed that participants who were continuously high actives and with high sedentary breaks from baseline to follow-up were less likely to have HBP, even after mutual adjustment for lifestyle behaviors.

The present study observed a wide increase in high screen-based sedentary behavior (17.2 vs 31.7) after 2 years of follow-up. The screen-based sedentary behavior corresponds to activities with low energy expenditure and has a different construct than physical activity, where individuals can reach global recommendations of physical activity and have high levels of screen-based sedentary time in the same day [30]. The substantial increase in screen-based sedentary time in the present study may be related to the fast technological advancements of screen devices, mainly regarding smartphones, with increasing capacity and functionality, as well as mutual interaction between devices and internet, allowing multiple tasks of daily life, such as occupational, educational, and entertainment activities, as well as popularization of instant messaging apps, online streaming services, and different platforms of social networks at the period of data collection in Brazil. It was also observed in the present study that adults with high levels of screen-based sedentary time were significantly younger than those with low levels (42.1 vs 63.2 years at baseline and 48.1 vs 67.1 years at follow-up). In this sense, it is possible that younger adults were more exposed to screen-based devices in daily life for occupational and entertainment activities than older adults, through increase mainly in cellphone time, besides computer and television.

It was also observed a slightly increase in the prevalence of overweight/obesity (67.7% vs 71.3%) after 2-year follow-up in this study sample. This increase may be related, among other factors, to the increase in sedentary behavior of the sample, since previous findings reported that high levels of sedentary behavior have been associated with overweight/obesity [31] and Duncan et al [32] observed that the proportion of people who engage in high screen time have increased in the same rate than those who were overweight or obese. In this sense, the reduction in daily energy expenditure caused by higher amount of sedentary activities may lead to a positive caloric imbalance and consequently weight gain. In addition, some studies have shown that high screen-based sedentary behavior has been associated with higher consumption of high-calorie-density foods, which could contribute to an increase in overweight/obesity rate [33, 34]. The eating behavior and its change from baseline to follow-up could help to better understand the present study findings, however they were not assessed in the present study and are recommended for future investigations. Another important factor that may have contributed to the increase of overweight/obesity in the sample was the high prevalence of participants who remained less physically active from baseline to follow-up (72% of sample), where physical activity engagement can be a mitigating factor of the deleterious effect of high sedentary time and contributing to weight control [35].

Adults who reported high sedentary breaks was less likely to have HBP both at baseline and follow-up, regardless of age, sex, socioeconomic status, and body mass index. The fragmentation of sedentary time has been associated to reduction in all-cause mortality and cardiovascular health of adults [36, 37], as well as better metabolic parameters [38]. This study finding corroborated with previous study by Loprinzi et al. [39], which reported that adults who spent more time in light intensity physical activities showed better cardiometabolic parameters than those with more prolonged sedentary behavior. The frequency of sedentary breaks resulted in an increase of overall physical activity levels [40] and consequently higher energy expenditure in adults [41]. These factors could support the negative association of sedentary breaks with HBP observed in the present study. The continuous engagement in sedentary breaks may mitigate risk factors for cardiovascular health: overweight, low physical activity, and prolonged sedentary behavior. As observed in high screen time group, participants with high sedentary breaks were younger than those with low sedentary breaks (56.3 vs 67.6 at baseline and 58.4 vs 69.4 at follow-up). A possible explanation is that participants with higher screen time may be more susceptible to fragmentate, precisely due to their higher amount, so that we observed that participants with high screen time were three times more likely to have high sedentary breaks (odds ratio 3.04, *p* < 0.001).

The present study also observed that adults who were continuously high actives from baseline to follow-up were less likely to have HBP. Physical activity has been widely reported as an important factor for high blood pressure prevention and monitoring [42, 43]. The mechanisms of physical activity for blood pressure lowering are diverse. Studies reported that physical activity contributes to reduction in peripheral vascular resistance and in arterial stiffness [44], where physical activity and inactivity have respectively vascular conditioning and deconditioning effects which can modify vasoconstrictor tone and arterial remodeling [45]. Other studies reported that physical activity was associated with higher parasympathetic modulation [46], and that adults who reached global recommendations for physical activity showed better relationship between cardiac autonomic modulation and cardiovascular parameters independently of overweight status [47]. Besides that, a reduction in the activity of renin-angiotensin-aldosterone system [48], an improvement in endothelial function [49], and improvement in hormonal imbalance of leptin and adiponectin [50] have also been reported as important mechanisms of physical activity effects on blood pressure lowering. Upon these findings, adults who were continuously high actives from baseline to follow-up possibly were exposed to acute and chronic contributions of physical activity over the time, resulting in a less chance of having HBP.

This study has important limitations. The assessment of physical activity, sedentary time, and sedentary breaks by questionnaire is susceptible to recall bias. The lack of cutoff points to define physically actives by Baecke questionnaire turn the score quartiles dependent on the level of physical activity from the sample and not on global recommendations. Future investigations with the use of accelerometer or other methods of physical activity evaluation such as heart rate recording are recommended for improvement of the results accuracy. In the other hand, the 2 years of follow-up, the randomly sample selection process, and adjustment of analysis by sociodemographic factors, body mass index, and mutually for the other lifestyle habits are important strengths of this study.

In conclusion, frequent sedentary breaks and high levels of physical activity over 2-year follow up were protective factor against HBP in middle-aged and older adults, while the worsening of lifestyle behavior showed to be harmful for cardiovascular health in community dwelling adults in a 2-year period. Future interventions for high blood pressure prevention and control needs to face lifestyle behaviors worsening in community environment. These interventions should be based on reducing the time in screen-based activities with low energy expenditure, encouraging of more frequent fragmentation of leisure sedentary time, and improvement of overall physical activity levels through different domains of daily life, such as active commuting, wider range of leisure time physical activities, and engagement sports practice, so that be possible to sustain these habits over time in adult population.

## Data Availability

The data underlying this article will be shared on reasonable request to the corresponding author.
